# Usefulness of hemoglobin A1c and glycated albumin measurements for insulinoma screening: an observational case-control study

**DOI:** 10.1186/s12885-019-5389-7

**Published:** 2019-02-26

**Authors:** Keiichi Torimoto, Yosuke Okada, Yoshiya Tanaka, Atsuko Matsuoka, Yushi Hirota, Wataru Ogawa, Yoshifumi Saisho, Isao Kurihara, Hiroshi Itoh, Shinya Inada, Masafumi Koga

**Affiliations:** 1 First Department of Internal Medicine, School of Medicine, University of Occupational and Environmental Health, Japan, 1-1 Iseigaoka, Yahatanishi-ku, Kitakyushu, Fukuoka, 807-8555 Japan; 2 Division of Diabetes and Endocrinology, Department of Internal Medicine, Kobe University Graduate School of Medicine, Hyogo, 650-0017 Japan; 3 Department of Internal Medicine, Keio University School of Medicine, Tokyo, 160-8582 Japan; 4 Department of Internal Medicine, Kawanishi City Hospital, Hyogo, 666-0195 Japan; 5 Department of Internal Medicine, Hakuhokai Central Hospital, Hyogo, 669-0953 Japan

**Keywords:** Insulinoma, HbA1c, Glycated albumin, Hypoglycemia

## Abstract

**Background:**

Insulinoma represents hypoglycemia as a predominant symptom; the autonomic symptoms may be resolved by chronically recurrent hypoglycemia resulting in the persistence of non-specific symptoms alone. Therefore, it has been estimated that there are many patients in whom the disease takes longer to diagnose and has remained undiagnosed. Although some parameters exist for the definitive diagnosis of the disease, there are currently no indices for early screening. Indices of glycemic control, hemoglobin A1c (HbA1c), and glycated albumin (GA) may be useful for the screening of patients with insulinoma having chronic hypoglycemia because the values become low in such a condition. Because there are no articles that have reported the point, we examine the effective cutoff values of HbA1c and GA for the diagnosis of insulinoma in the present study.

**Methods:**

In a multicenter cross-sectional study, 31 patients with insulinoma were included for comparison with 120 control subjects with normal glucose tolerance based on 75 g oral glucose tolerance tests whose characteristics were matched to the patients. The primary outcomes were optimal cutoff values of HbA1c and GA for the screening of insulinoma.

**Results:**

HbA1c was significantly lower in the insulinoma group at 4.7 ± 0.4% compared to the healthy control group at 5.7 ± 0.3% (*p* < 0.001), and GA was significantly lower in the insulinoma group at 11.6 ± 1.8% compared to the healthy control group at 14.5 ± 1.0% (p < 0.001). According to a receiver operating characteristic (ROC) analysis, optimal cutoff values of HbA1c and GA for the diagnosis of insulinoma were 5.0 and 12.4%, respectively. Area under the curve values of HbA1c and GA were 0.970 and 0.929, respectively, showing no significant difference (*p* = 0.399).

**Conclusions:**

In the present study, HbA1c and GA values in patients with insulinoma were significantly lower compared to the healthy controls, and effective cutoff values for screening were shown in the diagnosis of insulinoma for the first time. HbA1c and GA can be useful indices for insulinoma screening. Because malignant insulinoma have a similar diagnostic process to that of benign insulinoma, these could be useful for malignant insulinoma.

## Background

Insulinoma is a tumor derived from pancreatic beta cells that produce excessive insulin as the most common pancreatic neuroendocrine tumor. The predominant symptoms of the disease are autonomic and neurologic manifestations due to hypoglycemia. However, the autonomic symptoms accompanied by hypoglycemia may be resolved, and then only non-specific symptoms that do not seem to be hypoglycemic manifestations persist in patients with chronically recurrent hypoglycemia [1, 2]. For such a reason, it is suggested that the diagnosis may take at least 3.6 years in over half the patients [3], and many patients are often undiagnosed [4, 5]. Approximately 5–10% of insulinoma are malignant with distant metastasis at diagnosis [6], early detection is very important.

Classic diagnostic criteria of insulinoma are based on Whipple’s triad (hypoglycemic manifestation, plasma glucose level < 50 mg/dL, and improvement of the symptom with glucose intake); 72-h fasting and mixed diet tests have been performed for a definitive diagnosis [7]. However, there are no effective early screenings in patients with persistent non-specific symptoms alone.

Indices of glycemic control, hemoglobin A1c (HbA1c) and glycated albumin (GA) may be useful for screening patients with insulinoma having chronic hypoglycemia because the values become low in such a condition while no relevant information has been reported. In the present study, we examine the effective cutoff values of HbA1c and GA as indices of early screening for the diagnosis of insulinoma.

## Methods

### Study patients

Among patients with insulinoma who visited one of three medical institutions attending the multicenter study (Hospital of University of Occupational and Environmental Health, Kobe University Hospital, and Keio University Hospital), 31 patients who met the following inclusion criteria were included in the study. The inclusion criteria included (1) all ages and both sexes, (2) any HbA1c and GA values, and (3) benign insulinoma. Patients with malignant insulinoma, hepatic disease, renal disorder, and/or anemia were excluded from the study. We examined all patients between year 2000 and year 2016 with a diagnosis of insulinoma, non-metastatic with values for HbA1c or GA. Of the subjects in which 75 g oral glucose tolerance tests (75 g OGTT) were performed by a medical check, A total of 120 individuals with normal glucose level based on the World Health Organization criteria [8] whose characteristics [age, sex, and body mass index (BMI)] were matched to the patients above were included as a healthy control group [9]. This study observed the Declaration of Helsinki and current ethical codes and was approved by the ethics committee at the University of Occupational and Environmental Health. This research is not a clinical study, and the data was anonymized for retrospective analyses. Therefore, no written informed consent were obtained from the patients.

### Study outcomes

Blood tests were performed after overnight fasting. The age, sex, BMI, fasting plasma glucose (FPG), fasting immunoreactive insulin (IRI), HbA1c, and GA were investigated in both groups, and the primary outcomes were optimal cutoff values of HbA1c and GA for the screening of insulinoma.

### Measurements

Total of 120 individuals with normal glucose tolerance confirmed by 75 g OGTT were included in the study. Plasma glucose levels were measured by the hexokinase method. HbA1c, expressed as the National Glycohemoglobin Standardization Program value [10], was measured by high performance liquid chromatography. GA was determined by the enzymatic method using albumin-specific proteinase, ketoamine oxidase, and albumin assay reagent (Lucica GA-L; Asahi Kasei Pharma Co., Tokyo, Japan) [11].

### Statistical analyses

The values are indicated as mean ± standard deviation. Kolmogorov-Smirnov normality tests showed normal distributions for FPG and GA but not for age, BMI, HbA1c, and IRI. As for intergroup tests, unpaired t- and Mann-Whitney U tests were performed for data with a normal distribution and those without a normal distribution, respectively. In addition, chi-squared tests were conducted for comparisons between sexes. Receiver operating characteristic (ROC) curves were drawn to calculate areas under the curves and 95% confidence intervals for determination of the most distinguishable values of HbA1c and GA for the screening of insulinoma. For comparison of the diagnostic discrimination between HbA1c and GA, the areas under the curves (AUC) were compared by DeLong’s test. The significance level was *p* < 0.05. SPSS Statistical Software 25.0 (SPSS Inc., Chicago, IL) was used for the analyses.

## Results

The subject characteristics are shown in Table 1. The numbers of subjects in the insulinoma and healthy control groups were 31 (10 males and 21 females) and 120 (36 males and 84 females), respectively. No significant differences in age (58.3 ± 19.7 years vs. 57.3 ± 3.0 years; *p* = 0.608) and BMI (24.4 ± 4.3 kg/m^2^ vs. 23.9 ± 2.2 kg/m^2^; *p* = 0.390) were observed between the groups. FPG was significantly lower in the insulinoma group at 51.3 ± 15.3 mg/dL compared to the healthy control group at 93.0 ± 6.2 mg/dL (*p* < 0.001). On the other hand, fasting IRI was significantly higher in the insulinoma group at 17.0 ± 16.3 μU/mL compared to the healthy control group at 4.6 ± 2.1 μU/mL (*p* < 0.001).

**Table 1:** Comparison of clinical characteristics

	Insulinoma group (*n* = 31)	Control group (*n* = 120)	*P* value
Male gender, n (%)	10 (32.3)	36 (30.0)	0.808
Age, years	58.3 ± 19.7	57.3 ± 3.0	0.608
BMI, kg/m^2^	24.4 ± 4.3	23.9 ± 2.2	0.390
FPG, mg/dL	51.3 ± 15.3	93.0 ± 6.2	< 0.001
Fasting IRI, μU/mL	17.0 ± 16.3	4.6 ± 2.1	< 0.001
Fasting IRI/FPG	0.380 ± 0.433	0.050 ± 0.022	< 0.001
HbA1c, %	4.7 ± 0.4	5.7 ± 0.3	< 0.001
GA, %	11.6 ± 1.8	14.5 ± 1.0	< 0.001

Data are mean ± SD. BMI, body mass index; FPG, fasting plasma glucose; HbA1c, hemoglobin A1c; GA, glycate d albumin*. P* values are for differences between two groups

**Fig. 1:**
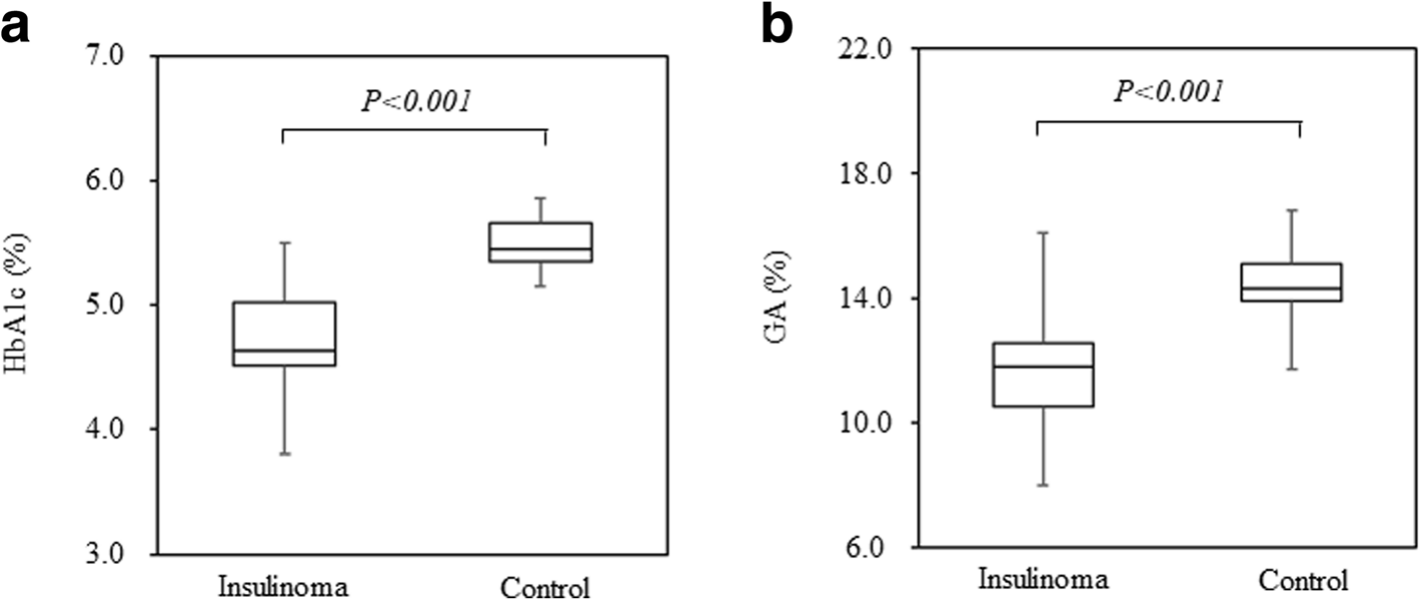
Comparison of HbA1c and GA between insulinoma and control. HbA1c (**a**) and GA (**b**) in insulinoma and control were shown. Data were compared by Student’s t-test for normally distributed variables and the Mann-Whitney U test for variables with a skewed distribution

HbA1c was significantly lower in the insulinoma group at 4.7 ± 0.4% compared to the healthy control group at 5.7 ± 0.3% (p < 0.001), and GA was significantly lower in the insulinoma group at 11.6 ± 1.8% compared to the healthy control group at 14.5 ± 1.0% [*p* < 0.001 (Fig. 1)].

**Fig. 2:**
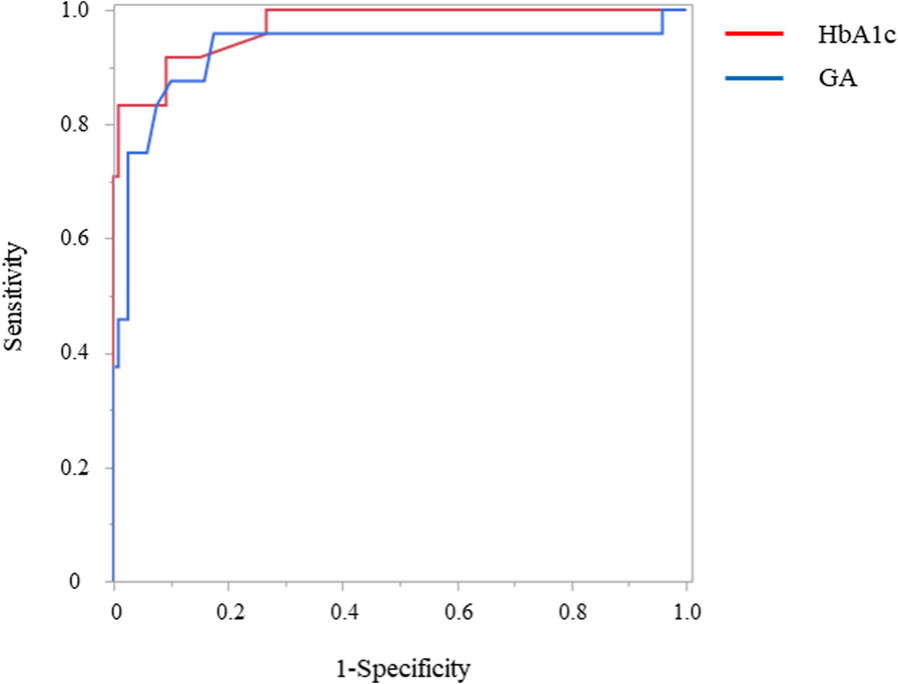
ROC curve of HbA1c and GA for predicting insulinoma. HbA1c: The optimal cutoff point of 5.0% (AUC = 0.971, 95% CI 0.918–0.990). GA: The optimal cutoff point of 12.4% (AUC = 0.929, 95% CI 0.788–0.979). ROC, receiver-operating characteristic; HbA1c, hemoglobin A1c; GA, glycated albumin, AUC; area under the curve

Then, ROC curves were drawn to calculate the AUCs and 95% confidence intervals for determination of the most distinguishable values of HbA1c and GA for the screening of insulinoma. The sensitivities, specificities, positive/negative predictive values, and positive/negative likelihood ratios by the cutoff value of HbA1c and GA are summarized in Table 2 and Table 3. The optimal cutoff value of HbA1c for the screening of insulinoma was determined to be 5.0% [AUC = 0.971; 95% CI, 0.918–0.990 (Table 2]). Moreover, the optimal cutoff value of GA for screening of insulinoma was determined to be 12.4% [AUC = 0.929; 95% CI, 0.788–0.979 (Table 3)]. AUC of HbA1c and GA were 0.970 and 0.929, respectively (Fig. 2), showing no significant difference (*p* = 0.221).

**Table 2:** Values for sensitivity, specificity positive predictive value, negative predictive value, positive likelihood ratio, and negative likelihood ratio for different cutoff points for HbA1c determined during screenings for insulinoma

HbA1c cut-off (%)	S (%)	Sp (%)	PPV (%)	NPV (%)	PLR	NLR
4.7	64.5	100.0	100.0	91.6	–	0.35
4.8	64.5	99.2	95.2	91.5	77.42	0.36
4.9	71.0	99.2	95.7	93.0	85.16	0.29
5.0	77.4	99.2	96.0	94.4	92.90	0.23
5.1	83.9	97.5	89.7	95.9	33.55	0.17
5.2	83.9	90.8	70.3	95.6	9.15	0.18
5.3	90.3	90.8	71.8	97.3	9.85	0.11
5.4	90.3	85.0	60.9	97.1	6.02	0.11
5.5	100.0	73.3	49.2	100.0	3.75	0

*S*, sensitivity; *Sp*, specificity; *PPV*, positive predictive value; *NPV*, negative predictive value; *PLR*, positive likelihood ratio; *NLR*, negative likelihood ratio

**Table 3:** Values for sensitivity, specificity positive predictive value, negative predictive value, positive likelihood ratio, and negative likelihood ratio for different cutoff points for GA determined during screenings for insulinoma

GA cut-off (%)	S (%)	Sp (%)	PPV (%)	NPV (%)	PLR	NLR
11.7	45.8	97.5	78.6	90.0	18.33	0.56
11.8	54.2	97.5	81.3	91.4	21.67	0.47
12.0	58.3	97.5	82.4	92.1	23.33	0.43
12.1	62.5	97.5	83.3	92.9	25.00	0.38
12.2	66.7	97.5	84.2	93.6	26.67	0.34
12.4	75.0	97.5	85.7	95.1	30.00	0.26
12.6	75.0	95.8	78.3	95.0	17.99	0.26
12.9	75.0	94.2	72.0	95.0	12.86	0.27
13.0	83.3	92.5	69.0	96.5	11.11	0.18
13.1	87.5	90.0	63.6	97.3	8.75	0.14
13.3	87.5	88.3	60.0	97.2	7.50	0.14
13.4	87.5	85.8	55.3	97.2	6.18	0.15
13.5	87.5	84.2	52.5	97.1	5.50	0.15
13.6	95.8	82.5	52.3	99.0	5.48	0.05
13.7	95.8	80.8	50	99.0	5.00	0.05

*S*, sensitivity; *Sp*, specificity; *PPV*, positive predictive value; *NPV*, negative predictive value; *PLR*, positive likelihood ratio; *NLR*, negative likelihood ratio

## Discussion

The present study indicated that HbA1c and GA values in patients with insulinoma were significantly lower than those in the healthy control by showing the effective cutoff values for the screening of insulinoma. Furthermore, AUCs in the ROC curves for screening of insulinoma were comparable between HbA1c and GA.

Insulinoma is a tumor, accounting for 70 to 80% of functional pancreatic neuroendocrine tumors, and most of the conditions are sporadic benign legions and single small highly vascular tumors [12]. The incidence of insulinoma in the general population was estimated to be one to four cases per million person-years [13]. In the meantime, the incidence was reported to be higher (0.8 to 10.0%) in some studies with autopsy, suggesting that insulinoma has been undiagnosed in many patients [4, 5]. The predominant symptoms of insulinoma include autonomic and neurologic manifestations due to hypoglycemia [14]; the autonomic symptoms accompanied by hypoglycemia may be resolved, and then only non-specific symptoms that do not seem to be hypoglycemic manifestations persist in patients with chronically recurrent hypoglycemia [1, 2]. Long-term exposure to chronic hypoglycemia may result in adaptation of the central nerve system to chronic hypoglycemia and lead to difficulty in recognizing hypoglycemia in most patients. In such cases, asymptomatic hypoglycemia can cause the loss of symptoms even with very low plasma glucose [15]. Symptoms of fasting hypoglycemia have occurred in 73% of patients with insulinoma. However, the residual 27% of patients have developed symptoms in both the fasting state and after meals or only after meals [16], and therefore detection of the symptoms can be delayed, especially in those cases. No screening methods have been reported for insulinoma. The present study showed that HbA1c and GA could be useful as parameters in the early screening for the diagnosis of insulinoma for the first time.

Whipple’s triad has been known as findings that can be suspected with insulinoma (presyncope in the fasting state, plasma glucose level < 50 mg/dL during attacks, and improvement of the symptom with glucose intake). Hypoglycemia has usually developed with autonomic regulation of insulin secretion in the fasting state, and fasting IRI measurement is therefore required for the diagnosis. In the present study, AUCs in the ROC curves of HbA1c and GA were similarly high in diagnoses of insulinoma, indicating that the precision of HbA1c and GA was as high as the screening tests.

Approximately 5–10% of insulinoma are malignant with distant metastasis at diagnosis [6], early detection is very important. The main clinical manifestations of malignant insulinoma are hypoglycemia-related symptoms and some malignant insulinoma tumors are diagnosed as benign pancreatic tumors [17]. This study didn’t include malignant insulinoma. Because malignant insulinoma have a similar diagnostic process to that of benign insulinoma [17], HbA1c and GA could be useful for malignant insulinoma screening.

This study had some limitations. The first was that the controls were individuals with normal glucose level. Those parameters could be good indices for screening, but should be investigated in subjects with hypoglycemia if used for the diagnosis of insulinoma. The second was that this research was a retrospective study with a small sample size. Further investigation of the results obtained from this study in a larger prospective cohort study is warranted.

## Conclusions

In the present study, HbA1c and GA values in patients with insulinoma were significantly lower compared to the healthy controls, and effective cutoff values for screening were shown in the diagnosis of insulinoma for the first time. HbA1c and GA can be useful indices for insulinoma screening in patients with chronic hypoglycemia.

## Abbreviations

75 g OGTT: 75 g oral glucose tolerance tests
AUC: Areas under the curves
BMI: Body mass index
FPG: Fasting plasma glucose
GA: Glycated albumin
HbA1c: Hemoglobin A1c
IRI: Immunoreactive insulin
ROC: Receiver operating characteristic
